# Anti-Ha Antisynthetase Syndrome: A Case Report

**DOI:** 10.7759/cureus.61251

**Published:** 2024-05-28

**Authors:** Vanessa P De Andrade, Renata Miossi, Fernando H De Souza, Samuel K Shinjo

**Affiliations:** 1 Rheumatology, Hospital das Clínicas da Faculdade de Medicina da Universidade de São Paulo (HCFMUSP), São Paulo, BRA

**Keywords:** case report, autoimmune rheumatic disease, rheumatic disease, inflammatory myopathy, antisythetase syndrome

## Abstract

Anti-synthetase syndrome (ASyS) is a rare systemic autoimmune myopathy characterized by the involvement of muscles, lungs, and joints, in addition to Raynaud’s phenomenon, “mechanics’ hand,” and fever. Laboratory ASyS is defined by the positivity of anti-aminoacyl-tRNA synthetase autoantibodies, of which anti-Jo-1 is the most common. Herein, we reported an ASyS defined by an anti-Ha autoantibody, which has rarely been described in the literature. Moreover, to the best of our knowledge, we reported the first case of anti-Ha ASyS in Brazil.

## Introduction

Anti-synthetase syndrome (ASyS) is a systemic autoimmune myopathy (or idiopathic inflammatory myopathy) characterized by the positivity of anti-aminoacyl-tRNA synthetase (ARS) autoantibodies and by the occurrence of a broad spectrum of clinical features of inflammatory myopathy, interstitial lung disease, non-erosive arthritis, Raynaud’s phenomenon, “mechanics’ hand,” and fever [1-4]. The most common anti-ARS autoantibodies are anti-Jo-1 (histidyl), followed by anti-PL-7 (threonyl), anti-PL-12 (alanyl), anti-EJ (glycyl), anti-OJ (isoleucyl), anti-KS (asparaginyl), anti-Zo (phenylalanyl), and anti-Ha (tyrosyl) [5]. However, these last two have rarely been described in the literature [6,7]. Therefore, we reported the first Brazilian patient with anti-Ha ASyS.

## Case presentation

A 50-year-old male smoker started experiencing weight loss, myalgia, and progressive, symmetrical, and predominantly proximal muscle weakness of the limbs. In 2009, the patient developed progressive dyspnea and Raynaud’s phenomenon. After five months, the patient was admitted to our hospital with a decline in overall condition and desaturation in room air (SaO2 86%), globally reduced muscle strength (Medical Research Council scale for muscle strength [8] of IV and V in proximal and distal limb muscles, respectively), and the presence of a nonpruritic, hyperkeratotic, and scaly eruption on the radial side of the hands. There was no history of fever, arthralgia, or arthritis.

**Figure 1:**
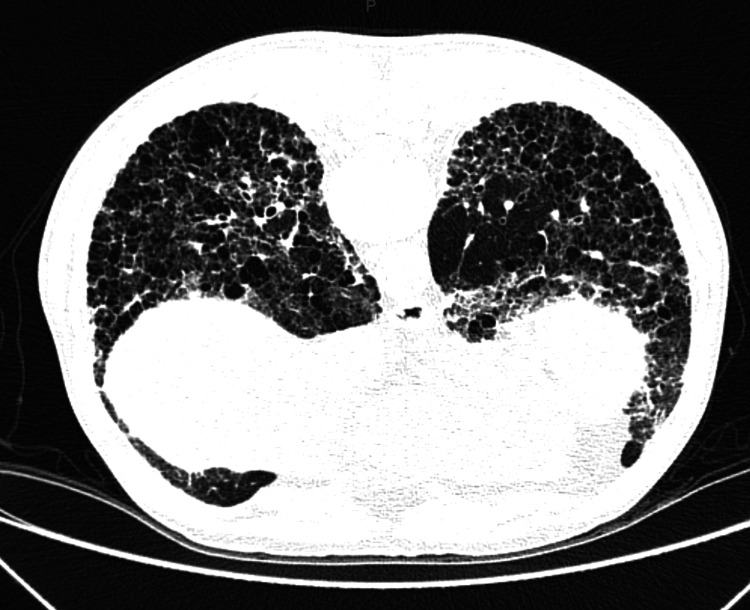
Chest high-resolution computed tomography.

**Figure 2:**
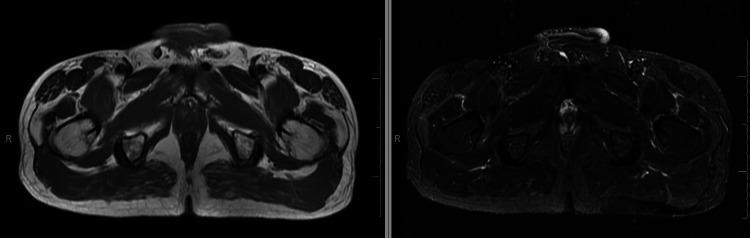
Muscle magnetic resonance imaging of the proximal part of both thighs. Left figure: Axial T1 image. Right figure: STIR image.

Radiological imaging of the chest (high-resolution computed tomography) revealed evidence of centriolobular and paraseptal pulmonary emphysema disseminated throughout all lung fields; diffuse thickening of bronchial walls, likely chronic and inflammatory, possibly related to a history of smoking; and diffuse infiltrate characterized by fine reticular opacities and ground-glass opacities associated with honeycombing cysts, bronchiectasis, traction bronchiectasis, and architectural distortion with volumetric reduction, bilaterally at the lung bases (Figure 1). Pulmonary function test with forced vital capacity (FVC) of 3.32 (71%), forced expiratory volume exhaled in the first second (FEV1) of 2.49 (66%), and FVC/FEV1 ratio of 0.75, with no response to bronchodilators. Transthoracic echocardiography showed a pulmonary artery systolic pressure of 55 mmHg, right ventricular dysfunction, hypokinesia, and diastolic dysfunction type 1. Nailfold capillaroscopy revealed an SD pattern. Finally, thigh muscle magnetic resonance imaging indicated mild diffuse muscle edema in all muscle compartments (Figure 2).

Laboratory tests showed a creatine phosphokinase (CPK) of 3539 U/L (reference range: 30-200 U/L), a positive antinuclear antibody with a cytoplasmic pattern of 1/320; myositis-specific autoantibodies (anti-Jo-1, anti-OJ, anti-EJ, anti-PL-7, anti-PL-12, anti-Mi-2, and anti-SRP) and myositis-associated autoantibodies (anti-PM/Scl75, anti-Ku, and anti-PM/Scl100) were negative, except for a positive anti-Ro52, according to a commercially available line blot test kit (Myositis Profile Euroline Blot test kit, Euroimmun, Lübeck, Germany). The assessment was performed according to previously established methods [8].

With a possible diagnosis of ASyS, even before autoantibody results, treatment was initiated with prednisone 1 mg/kg/day in addition to azathioprine 2.5 mg/kg/day. Subsequently, there was a complete improvement in muscle weakness, normalization of serum CPK levels, and stabilization of the pulmonary condition, allowing for progressive reduction and eventual discontinuation of prednisone.

More recently, the patient’s serum sample collected during the initial investigation was re-evaluated and confirmed the negativity of the previously mentioned autoantibodies. However, other anti-ARS autoantibodies (anti-Ha, anti-Zo, and anti-KS) were assessed, which tested positive for anti-Ha autoantibody (69.1 U, reference value < 10 U; value more than > 20 U), according to the method previously established [9]. Therefore, we concluded that the patient was ASyS-anti-Ha positive.

Currently, the patient has stable disease from a clinical and laboratory point of view and has been only using azathioprine 2.5 mg/kg/day.

## Discussion

To the best of our knowledge, this is the first case of anti-Ha ASyS in Brazil. This disease is characterized by a combination of symptoms such as myositis, non-erosive arthritis, interstitial lung disease, Raynaud’s phenomenon, “mechanic’s hands,” and fever. However, different patients may present with distinct clinical presentations [10], and these autoantibodies can influence the clinical presentation.

According to the Euromyositis Registry, patients with ASyS most often suffer from myositis and interstitial lung disease [11], and in cases of anti-Ha antibody-positive interstitial lung disease, most subjects exhibit preserved pulmonary function [12]. However, in the present case, the patient had a history of smoking, which may have directly influenced the pulmonary function.

In the literature, anti-Ha ASyS [8] demonstrated a favorable response to pharmacological treatment, which may reveal a more favorable prognosis in these cases. Consistent with these studies, our patient showed an excellent response to therapy instituted with rapid weaning from prednisone, which may also indicate a subgroup among patients with ASyS with a better global prognosis in addition to lung disease.

## Conclusions

In clinical practice, it is important to consider ASyS when patients have classic involvement of the lungs, muscles, and/or joints. The presence of other symptoms and signs, such as Raynaud’s phenomenon, fever, and “mechanics’ hand,” corroborated the hypothesis of ASyS. In these cases, the assessment of anti-ARS autoantibodies is essential to confirm the diagnosis. However, negativity toward principals would not rule out ASyS. In the present case, we conducted anti-synthetase syndrome (ASyS) autoantibody testing despite the primary autoantibodies yielding negative results. Subsequently, a definitive diagnosis was established.
